# Highly variable trends in rates of newly diagnosed HIV cases in U.S. hotspots, 2008-2017

**DOI:** 10.1371/journal.pone.0250179

**Published:** 2021-04-19

**Authors:** Lorena Segarra, Samuel J. Simmens, Amanda D. Castel, Michael Kharfen, Henry Masur, Alan E. Greenberg

**Affiliations:** 1 Department of Epidemiology, George Washington University Milken Institute School of Public Health, Washington, DC, United States of America; 2 Department of Biostatistics and Bioinformatics, George Washington University Milken Institute School of Public Health, Washington, DC, United States of America; 3 HIV/AIDS, Hepatitis, STD & TB Administration, DC Department of Health, Washington, DC, United States of America; 4 National Institutes of Health, Bethesda, Maryland, United States of America; Chang Gung Memorial Hospital and Chang Gung University, Taoyuan, TAIWAN

## Abstract

The U.S. Ending the HIV Epidemic (EHE) initiative was announced in early 2019 and rapidly became a focal point for domestic HIV prevention and treatment programs. Using publicly available data from CDC, we examined historical trends in the average annual percent change (AAPC) in HIV diagnosis rates for the 57 EHE high incidence “hotspots” using Joinpoint analysis. We then assessed the ecologic association of various hotspot characteristics with changes in these rates over time using a multivariable regression model. From 2008–2017, the overall rate of newly diagnosed HIV cases in the U.S. declined from 19 to 14 per 100,000 persons, with the AAPC declining significantly in the U.S. overall (-3.1%; 95% CI: -3.7, -2.4) and in the 57 hotspots (-3.3%; 95% CI: -4.6, -2.8). There were large (AAPC <-5.0), moderate (-5.0 to -2.5) and small (-2.5 to 0.0) rates of decline in 14, 19 and 17 hotspots respectively, with increasing trends (AAPC >0.0) noted in seven hotspots. In the multivariable regression analysis, higher initial HIV diagnosis rate and location in the Northeast region were significantly associated with declining AAPC rates whereas no significant differences were found by hotspot gender, age, or race/ethnicity distribution. This analysis demonstrates that the rate of decline in HIV diagnosis rates in hotspots across the U.S. has been highly variable. Further exploration is warranted to assess the correlation between programmatic factors such as HIV testing and antiretroviral therapy and pre-exposure prophylaxis coverage with HIV trends across the hotspots.

## Introduction

Four decades have passed since the first cases of what would become known as the acquired immunodeficiency syndrome (AIDS) were reported in 1981 [1]. Globally, there are currently 38 million persons living with the human immunodeficiency virus (HIV), the virus that causes AIDS, and there were 690,000 AIDS-related deaths and 1.7 million new HIV infections in 2019 [2]. In the United States, 1.2 million persons are living with diagnosed HIV infection, and there were 38,000 new HIV-related diagnoses and 16,000 deaths in 2018 [3]. Despite noteworthy advances in the development and dissemination of antiretroviral medications used for HIV treatment [4], treatment as prevention (TasP) [5] and pre-exposure prophylaxis (PrEP) [6], the estimated HIV incidence in the U.S. remained between 36,400 and 38,500 from 2014–2018 [7].

In early 2019, the United States Department of Health and Human Services (HHS) launched an initiative to end the HIV epidemic (EHE) in the United States by 2030 [8]. This initiative included four key strategies namely early HIV diagnosis, rapid and effective use of antiretroviral therapy, prevention interventions including pre-exposure prophylaxis and rapid identification of and response to HIV outbreaks [9–11]. The EHE initiative initially focused on 57 “hotspots”– 48 counties across 19 states, Washington, DC, San Juan, Puerto Rico, and seven states with a high rural HIV burden—that together accounted for more than 50% of newly diagnosed HIV cases in the past several years [8,11,12]. Phase I of the plan included the provision of federal funds to the National Institutes of Health (NIH) for HIV implementation science research to help guide the strategic process [13,14].

Despite the considerable promise of the EHE initiative, a substantial number of structural challenges and individual-level factors need to be addressed to ensure its success [15]. To help inform the EHE initiative, we examined trends in rates of newly diagnosed HIV cases in the 57 hotspots over the past decade and assessed the ecologic association of various hotspot characteristics with changes in these rates over time.

## Methods

Aggregate data on newly diagnosed HIV cases (age >= 13 years old) in the U.S. and in each hotspot from 2008–2017, and population denominators based on data from the U.S. Census Bureau were obtained from the U.S. Centers for Disease Control and Prevention AtlasPlus website [16]. AtlasPlus is an online, interactive tool that uses publicly available national HIV surveillance data to create tables, graphs and maps. Each hotspot was assigned to a region using the U.S. Census Bureau regional classification system [17].

Statistical analyses of trends were conducted using Joinpoint Trend Analysis Software Version 4.7.0.0 which was developed by the National Cancer Institute to estimate and test trends in cancer rates and to identify changes in trends or “joinpoints” using a grid-search method with a Monte Carlo permutation method to test for significance [18]. This analytic approach has been used recently to study trends in racial/ethnic disparities of new AIDS diagnoses, HIV diagnoses among men who have sex with men and pre-exposure prophylaxis coverage [19–21]. Trend lines are summarized through two statistics with nearly identical interpretations: 1) the Annual Percent Change (APC), which represents the estimated percent change in incidence each year for a single linear trend line; and 2) the Average Annual Percent Change (AAPC), which is a weighted average of APC statistics when there is more than one linear trend line (e.g., one joinpoint) for a hotspot. In this paper, we follow the Jointpoint software convention of labeling the trend of the full 10-year period as the AAPC, although it is equivalent to the APC statistic when there is no joinpoint. Software options were selected to use heteroscedastic standard errors incorporated into weighted log-linear regression of the trend lines. This allowed standard errors to vary over time and assumes an annual percent change in incidence rather than a constant change in absolute incidence. Although it is plausible that a hotspot could experience multiple shifts in incidence trend over a 10 year period, the software was constrained to estimate at most one joinpoint per hotspot due to the potential unreliability in estimating those joinpoints when there are very few years of data per segment. HIV diagnosis and population data for each hotspot and the U.S. was imported to calculate crude HIV diagnosis rates per 100,000. P-values for the 57 10-year hotspot AAPC trends were adjusted for multiple testing through the Hochberg approach [22] and an adjusted p-value < .05 was considered statistically significant. For hotspots that had evidence of two distinct rate trends during the 10-year study period (i.e. those with a significant joinpoint), the APC of HIV diagnosis rates was estimated for each time period.

To facilitate the graphic presentation and visualization of trends data from a large number of sites, hotspot trends are presented together and then stratified into four categories: those with the largest rates of decline in AAPC (<-5.0), moderate rates of decline in AAPC (-5.0 to -2.5), smallest rates of decline in AAPC (-2.5 to 0.0), and increases in AAPC (>0.0). The absolute difference between the number of HIV diagnoses at the start (2008) and end (2017) of the 10-year analytic period was calculated for each hotspot.

A multivariable weighted linear regression model was developed to examine the association of various hotspot characteristics with the hotspot AAPC. These characteristics include ecological variables representing the demographic mix of newly diagnosed HIV cases (proportion female, proportion in different race/ethnic groups, and proportion over 35 years old), as well as geographic region and rate of new HIV diagnoses in 2008. County-level data on programmatic factors such as HIV treatment and PrEP coverage were available on the AtlasPlus website from 2017–2018 [16]; since our HIV trends analysis spanned the period 2008–2017, these periods were non-overlapping and thus we did not include these factors as covariates in this analysis.

The AAPC for each hotspot over the full 10-year period was the dependent variable and the presence of possible joinpoints was ignored for this analysis. Hotspot gender and race/ethnicity variables were calculated as the proportion of cases over the 10-year period within demographic categories, e.g., proportion male. Because of mathematical constraints, proportion female and proportion White were not included in the model and serve as reference variables similar to a regression reference category for nominal variables. In addition, associations with AAPC were estimated through Pearson correlations and simple linear regression. Because larger hotspots generally have more precise AAPC estimates and therefore violate the homoscedasticity assumption of linear regression, Pearson correlations and regression models were weighted by the relative total number of cases per hotspot. Consequently, larger hotspots have a greater influence on these analyses. Six hotspots had missing data for at least one variable and thus the sample sizes for Pearson Correlations and simple linear regressions ranged from 51 to 57; these hotspots were not included in the multivariable regression model. Analyses were conducted using SAS 9.4, SAS/STAT version 15.1.

## Results

**Fig 1:**
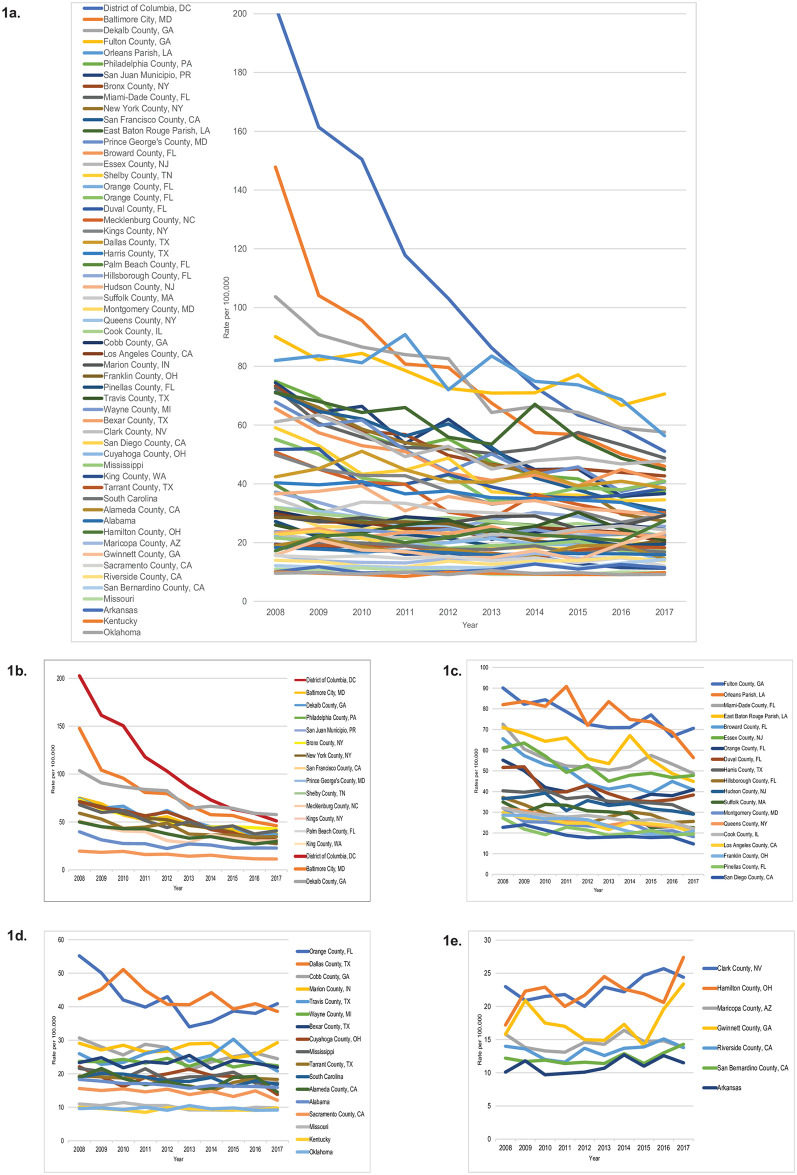
Rates per 100,000 of newly diagnosed HIV cases in US hotspots, 2008–2017. a. All hotspots. b. Hotspots with largest declines in AAPC (<-5). c. Hotspots with moderate declines in AAPC (-5 to -2.5). d. Hotspots with smallest declines in AAPC (-2.5 to 0.0). e. Hotspots with increasing AAPC (>0.0). Footnote. The scale of the y axis differs in each graph to enhance the visualization of differences in trends.

From 2008–2017, the overall rate of newly diagnosed HIV cases in the U.S. declined from 19 to 14 per 100,000 persons, with the AAPC declining significantly in both the overall U.S. population (-3.1%) and the pooled data from the 57 hotspots (-3.3%) (Table 1). The AAPC declined in 51 hotspots (89.5%) of which 21 (41.2%) declined significantly and increased in 6 hotspots (10.5%) of which none increased significantly (Table 1 and Fig 1a).

**Table 1:** Average annual percent change (AAPC) and change in number of newly diagnosed HIV cases in US hotspots, 2008–2017.

			**AAPC %**	**95%CI**	
**United States**			-3.1*	-3.7, -2.4	
**All Hotspots Combined**			-3.7*	-4.6, -2.8	
**Hotspots**	**Urban Center**	**Region**	**AAPC %**	**95%CI**	**Change in No. HIV Cases**
**Largest Declines in AAPC (<-5.0)**					
District of Columbia, DC	Washington	South	-14.5*	-15.6, -13.5	-724
Baltimore City, MD	Baltimore	South	-11.9*	-14.0, -9.9	-551
New York County, NY	New York	Northeast	-10.2*	-12.2, -8.2	-624
San Francisco County, CA	San Francisco	West	-8.9*	-10.5, -7.3	-269
Philadelphia County, PA	Philadelphia	Northeast	-7.8*	-9.2, -6.4	-459
San Juan Municipio, PR	San Juan	-	-7.5*	-9.3, -5.6	-158
Dekalb County, GA	Atlanta	South	-6.4*	-7.6, -5.1	-272
Kings County, NY	New York	Northeast	-6.3*	-7.5, -5.1	-405
King County, WA	Seattle	West	-6.0*	-7.4, -4.5	-98
Palm Beach County, FL	W Palm Beach	South	-6.0	-10.5, -1.2	-140
Prince George’s County, MD	Bowie	South	-5.9*	-7.7, -4.0	-154
Shelby County, TN	Memphis	South	-5.9*	-7.8, -3.9	-171
Bronx County, NY	New York	Northeast	-5.5*	-7.0, -6.6	-313
Mecklenburg County, NC	Charlotte	South	-5.4	-8.0, -2.7	-96
**Moderate Declines in AAPC (-5.0 to -2.5)**					
Franklin County, OH	Columbus	Midwest	-4.9*	-6.5, -3.3	-43
Suffolk County, MA	Boston	Northeast	-4.7*	-6.7, -2.6	-66
Broward County, FL	Fort Lauderdale	South	-4.6*	-7.1, -2.0	-288
Montgomery County, MD	Rockville	South	-4.4*	-6.2, -2.6	-92
San Diego County, CA	San Diego	West	-4.1*	-5.8, -2.4	-153
E Baton Rouge Parish, LA	Baton Rouge	South	-4.1	-6.3, -1.8	-87
Queens County, NY	New York	Northeast	-4.0*	-5.5, -2.6	-184
Duval County, FL	Jacksonville	South	-3.9	-5.9, -1.7	-61
Miami-Dade County, FL	Miami	South	-3.6	-7.1,0.1	-354
Essex County, NJ	Newark	Northeast	-3.3	-5.0, -1.6	-68
Orleans Parish, LA	New Orleans	South	-3.2	-5.5, -0.9	-45
Hillsborough County, FL	Tampa	South	-3.1	-5.5, -0.7	-58
Orange County, FL	Orlando	South	-3.1	-6.4,0.3	-22
Cook County, IL	Chicago	Midwest	-3.0*	-4.0, -2.1	-415
Los Angeles County, CA	Los Angeles	West	-3.0	-4.6, -1.5	-667
Harris County, TX	Houston	South	-3.0*	-3.9, -2.0	-164
Fulton County, GA	Atlanta	South	-2.7	-3.9, -1.4	-133
Hudson County, NJ	Jersey City	Northeast	-2.6	-4.2, -1.0	-19
Pinellas County, FL	St. Petersburg	South	-2.6	-4.8, -0.4	-48
**Hotspots**	**Urban Center**	**Region**	**AAPC %**	**95%CI**	**Change in No. HIV Cases**
**Smallest Declines in AAPC (-2.5 to 0.0)**					
Cobb County, GA	Atlanta	South	-2.1	-4.2, 0.1	-21
Cuyahoga County, OH	Cleveland	Midwest	-2.0	-4.9, 1.1	-91
Alameda County, CA	Oakland	West	-2.0	-4.6, 0.8	-27
Mississippi		South	-1.9	-3.5, -0.2	-90
Sacramento County, CA	Sacramento	West	-1.8	-3.3, -0.3	-23
Dallas County, TX	Dallas	South	-1.8	-3.6, 0.0	10
Missouri		Midwest	-1.8	-3.2, -0.3	-40
South Carolina		South	-1.8	-2.9, -0.6	2
Alabama		South	-1.4*	-1.8, -0.9	-58
Wayne County, MI	Detroit	Midwest	-0.7	-1.6,0.3	-53
Tarrant County, TX	Fort Worth	South	-0.5	-2.5, 1.5	39
Bexar County, TX	San Antonio	South	-0.4	-1.9, 1.0	50
Oklahoma		South	-0.4	-1.6,0.9	12
Marion County, IN	Indianapolis	Midwest	-0.3	-1.9,1.3	19
Orange County, CA	Los Angeles	West	-0.3	-2.5,1.8	-11
Kentucky		South	-0.2	-1.4,1.1	10
Travis County, TX	Austin	South	-0.1	-3.1,2.9	2
**Increases in AAPC (>0.0)**					
Maricopa County, AZ	Phoenix	West	0.2	-1.7,2.1	-3
Riverside County, CA	Los Angeles	West	1.1	-0.7,3.0	42
Clark County, NV	Las Vegas	West	1.8	0.3, 3.4	95
Arkansas		South	1.8	-0.3, 4.1	49
San Bernardino County, CA	Los Angeles	West	2.2	-1.0,5.4	57
Hamilton County, OH	Cincinnati	Midwest	2.4	-0.3, 5.2	64
Gwinnett County, GA	Atlanta	South	2.9	-4.1, 10.4	77

*Adjusted p-value< 0.05 using the Hochberg adjustment for multiple comparisons.

AAPC = Average annual percent change.

The hotspots with the largest declines in HIV diagnosis rates with an AAPC of <-5.0 are shown in Table 1 and Fig 1b. The 10 hotspots with the steepest declines were the District of Columbia, Baltimore City, New York County, San Francisco County, Philadelphia County, San Juan Municipio, Dekalb County, Kings County, King County and Palm Beach County. Hotspots with moderate declines in HIV diagnosis rates with an AAPC of -5.0 to -2.5 are shown in Table 1 and Fig 1c; hotspots with the smallest declines in HIV diagnosis rates with an AAPC of -2.5 to 0.0 are shown in Table 1 and Fig 1d; and hotspots with increases in HIV diagnosis rates with an AAPC of >0.0 are shown in Table 1 and Fig 1e.

The change in the number of new HIV diagnoses in the hotspots from 2008 to 2017 is shown in the rightmost column in Table 1. Overall, 43 hotspots had a net decrease and 14 had a net increase in the number of HIV diagnoses. The 10 hotspots with greatest absolute declines in the number of HIV diagnoses were the District of Columbia and Los Angeles, New York, Baltimore City, Philadelphia, Cook, Kings, Miami-Dade, Bronx and Broward Counties. Six of these hotspots were in the group with the largest declines in AAPC, and four were in the group with moderate declines in AAPC. The largest declines in the number of new HIV diagnoses clustered geographically in Washington, DC, Baltimore, Philadelphia and New York City; Miami and Fort Lauderdale; and Atlanta, Chicago, Los Angeles and San Francisco.

Nine hotspots had significant joinpoints (Table 2), indicating evidence of two distinct rate trends over the ten-year period. Of these, there were three with a significantly declining rate followed by a significantly declining but lower rate (Baltimore City, Bronx and Queens Counties), two with a significantly declining rate followed by a significantly declining but higher rate (New York and San Francisco Counties), and four with a significantly declining rate followed by a flat or non-significantly increasing rate (Alabama and Broward, Orange and Tarrant Counties).

**Table 2:** Trends in US hotspots with significant changes in AAPC using Joinpoint regression analysis, 2008–2017.

Hotspots	AAPC %	95% CI	Time Period	APC %	95% CI
Baltimore City, MD	-11.9*	-14.0, -9.9	2008–2010	-20.1*	-28.7, -10.5
			2010–2017	-9.4*	-11.4, -7.5
New York County, NY	-10.2*	-12.2, -8.2	2008–2014	-7.7*	-9.5, -5.8
			2014–2017	-14.9*	-21.4, -8.0
San Francisco County, CA	-8.9*	-10.5, -7.3	2008–2012	-4.6*	-7.9, -1.1
			2012–2017	-12.2*	-14.8, -9.5
Bronx County, NY	-5.5*	-7.0, -6.6	2008–2013	-8.4*	-10.6, -6.2
			2013–2017	-1.7	-5.5, 2.2
Broward County, FL	-4.6*	-7.1, -2.0	2008–2013	-8.5*	-12.0, -4.8
			2013–2017	0.6	-5.3, 6.9
Queens County, NY	-4.0*	-5.5, -2.6	2008–2011	-7.2*	-11.4, -2.7
			2011–2017	-2.4*	-4.2, -0.7
Orange County, FL	-3.1	-6.4,0.3	2008–2013	-8.1*	-12.9, -3.2
			2013–2017	3.5	-4.2,11.8
Alabama	-1.4*`	-1.8, -0.9	2008–2013	-2.5*	-3.1, -1.8
			2013–2017	0.0	-1.0, 1.1
Tarrant County, TX	-0.5	-2.5, 1.5	2008–2014	-3.7*	-5.9, -1.5
			2014–2017	6.2	-0.4,13.3

* Unadjusted p-value< 0.05.

APC = Annual percent change.

AAPC = Average annual percent change.

Unadjusted and multivariable adjusted associations of hotspot characteristics with AAPC are shown in Table 3. HIV diagnosis rate in 2008 was strongly associated with decreasing AAPC (r = -0.81, p < .001; partial correlation = -0.72, p < .001). That is, the higher the HIV diagnosis rate in 2008, the greater the decline in rate over the next 10 years. The Northeast showed a steeper decline in the HIV diagnosis rate than other regions in both the unadjusted association and in the multivariable model. The regression coefficient of -2.17 (p < .05) indicates that the mean AAPC in the Northeast was 2.17 points lower than the South after controlling for the other model variables. The South had the lowest overall decline among regions despite the fact that the two hotspots with the greatest declines, Washington, DC and Baltimore, were classified as part of the South. Other variables showing unadjusted associations with decreasing AAPC were hotspot age under 35, proportion female, proportion Black, and proportion not White, but these associations were not statistically significant in the multivariable analysis.

**Table 3:** Multivariate regression model to assess the association of hotspot characteristics with 10-year AAPC trends.

Covariate		Multivariable Model (N = 51) ^c^	
	Pearson Correlations	Partial Correlations	Regression Coefficients (95% CI)
**Region** ^a^			
**Northeast**	-0.36*	-0.40*	-2.17* **(-3.74, -0.61)**
**West**	**0.20**	**-0.01**	**-0.08 (-1.96, 1.79)**
**Midwest**	**0.17**	**-0.15**	**-0.76 (-2.42, 0.87)**
**South**	**0.01**	**ref**	**ref**
**HIV Rate in 2008**	-0.81***	-0.72***	-0.07*** **(-0.09, -0.05)**
**Proportion 35 and older**	-0.51***	**-0.21**	**-9.85 (-23.99, 4.30)**
**Proportion female**	-0.30*	**-0.03**	**-1.39 (-14.63, 17.41)**
**Race/Ethnicity proportions** ^b^			
**Hispanic/Latino**	**0.04**	**0.16**	**2.83 (-2.71, 8.36)**
**Black**	-0.30*	**0.03**	**0.66 (-6.47, 7.79)**
**Asian/Other**	**-0.06**	**0.26**	**-16.75 (-36.54, 3.04)**
**White**	0.30*	**Ref**	**ref**

*p < .05,

**p < .01,

***p < .001.

^a^ Regions are dummy coded thus the Pearson correlations contrast each region to all other regions, while the partial correlations and regression coefficients contrast each region to the South.

^b^ Pearson correlations contrast each Race/Ethnicity group to all of groups, while the multivariable statistics contrast each Race/Ethnicity group to the White group.

^c^ San Juan was excluded from the multivariable model because of missing data.

## Discussion

The Ending the HIV Epidemic (EHE) initiative seeks to reduce the number of new HIV infections in the United States by 75 percent by 2025, and then by at least 90 percent by 2030. Assessment of trends over the 10-year period 2008–2017 demonstrated that prior to the EHE many of the identified “hotspots” had made substantial progress in reducing the number of new HIV diagnoses whereas others had not. Comparisons of the programmatic approaches taken in jurisdictions with varying outcomes in reducing new HIV diagnosis rates should provide lessons relevant to achieving the ambitious 2025 and 2030 goals. This analysis complements a recent comprehensive overview of the epidemiology of HIV in the US that highlighted regional disparities in the HIV epidemic with a geographic concentration in the South [23].

The 14 hotspots that achieved the steepest *rates* of decline in new HIV diagnoses as measured by AAPC included seven in the South (District of Columbia, Baltimore City, Prince George’s, Mecklenburg, Shelby, Palm Beach and DeKalb Counties), four in the Northeast (New York, Kings, Bronx and Philadelphia Counties), two in the West (San Francisco and King Counties) and San Juan Municipio. Each of these hotspots was also among the 22 hotspots with the largest decline in the *number* of annual HIV diagnoses. In the regression analysis, the strongest predictor of a declining AAPC among the hotspots was the initial rate of new HIV diagnoses in 2008. Some of the observed changes in trends could have been due to increased testing at the start of the observational period since we are assessing new HIV diagnoses and not HIV incidence; this could be explored by analyzing the proportion of delayed HIV diagnoses over time. In addition, although it is possible that regression to the mean may partly account for the observed changes in rates, the available data cannot be used to assess this. Another correlate of AAPC decline was being in the Northeast region with no significant correlation found between a declining AAPC and gender, race/ethnicity, or age proportions. Our regression model used ecological variables as predictors and consequently, these results might not apply to individual level predictors. Further exploration of HIV programs in jurisdictions with dramatic declines including HIV funding levels, HIV testing, antiretroviral treatment and pre-exposure prophylaxis coverage and other structural or policy interventions should provide insights that can inform program prioritization and scale-up for the national end the HIV epidemic initiative.

The seven hotspots that experienced an increase in the *rates* of new HIV diagnoses were located in the West (Maricopa, Riverside, Clark and San Bernardino Counties), the South (Arkansas and Gwinnett County) and the Midwest (Hamilton County); six of these were also among the eight hotspots with the greatest increase in the *number* of HIV diagnoses, along with Tarrant and Bexar Counties in Texas. As with the hotspots that experienced declines in rates, more in-depth analysis is needed to determine whether programmatic factors or demographic factors were associated with these increases.

The Joinpoint regression analysis was effective in identifying trends in AAPCs that had significant changes in slope during the study period. Moving forward, this technique can be applied to determine whether the EHE initiative will significantly “bend the curve” of new HIV diagnoses both nationally and in local jurisdictions.

This analysis has several compelling strengths including the use of national publicly available databases, the inclusion of data from all 57 hotspots, multicolor visualization to facilitate the interpretation of complex data and the use of Joinpoint and multivariable regression analyses. There were also several notable limitations including the lack of available data for several demographic parameters in some hotpots and risk factor information and programmatic data in all hotspots, and assessing trends concurrently at the state and county level.

In summary, the impact of strategies and investments in HIV prevention appear to have been highly variable across jurisdictions. Hotspots with the steepest declines in rates of new HIV diagnoses should be praised for their successes. Jurisdictions with less dramatic decreases often started with substantially lower rates—one relevant factor might be the increasing difficultly of reducing “low” rates to even lower rates. Moreover, the analytic techniques described in this paper could be applied to other geographic areas.

The establishment of baseline trends in HIV diagnosis rates across all EHE hotspots serves as a useful starting point from which the impact of the EHE initiative can be monitored prospectively. Furthermore, assessing the differential impact of the initiative among various hotspots will provide real-time data that can be used to inform decisions about the allocation of limited resources.
